# Carnivore Diet: A Scoping Review of the Current Evidence, Potential Benefits and Risks

**DOI:** 10.3390/nu18020348

**Published:** 2026-01-21

**Authors:** Almiera Lietz, Janina Dapprich, Tobias Fischer

**Affiliations:** Center for Nutrition and Therapy (NuT), University of Applied Sciences Muenster, Corrensstraße 25, 48149 Muenster, Germany

**Keywords:** carnivore diet, Lion Diet, plant-free diet, ketogenic diet, nutrient adequacy, cardiovascular risks, microbiome, sustainability, LCHF

## Abstract

**Background**: The Carnivore Diet (CD) is an almost exclusively animal-based dietary pattern that has gained increasing popularity on social media. Despite numerous health-related claims, a standardized definition is lacking, and scientific evidence regarding the long-term effects of this diet remains unclear. **Methods**: The literature search for this scoping review was conducted in accordance with PRISMA guidelines (PRISMA-ScR) using the databases PubMed, LIVIVO, Web of Science, and the Cochrane Library. **Results**: Nine human studies were included. Individual publications reported positive effects of the CD, such as weight reduction, increased satiety, and potential improvements in inflammatory or metabolic markers. At the same time, potential risks of nutrient deficiencies, particularly in vitamins C and D, calcium, magnesium, iodine, and dietary fiber, as well as elevated low-density-lipoprotein (LDL-) and total cholesterol (TC) levels, were identified, along with one case describing a deterioration in health status. Overall, the quality of evidence is very limited due to small sample sizes, short study durations, and the absence of control groups. **Conclusions**: The CD may offer short-term health benefits but carries substantial risks of nutrient deficiencies, reduced intake of health-promoting phytochemicals, and the development of cardiovascular disease. At this time, long-term adherence to a CD cannot be recommended.

## 1. Introduction

In recent years, a clear international shift toward plant-based dietary patterns has been observed [1]. Meanwhile, an exclusively animal-based dietary approach, the so-called Carnivore Diet (CD), has gained increasing attention, particularly on social media. For example, the hashtag #carnivore on Instagram features approximately 2.6 million posts (as of 28 November 2025). To date, no standardized definition of the CD exists. In general, the CD is characterized by the exclusive consumption of minimally processed animal-source foods, including meat (including organ meats), fish, seafood, eggs, animal fats, and varying amounts of full-fat dairy products. Plant-based foods are entirely eliminated, and both carbohydrates and highly processed products are largely avoided [2,3]. Red meat typically forms the basis of the diet; the strictest variant, known as the “Lion Diet,” consists solely of red meat, salt, and water [4]. Given its nutrient profile, the CD can be categorized within low-carbohydrate/high-fat (LCHF) dietary patterns. Depending on protein intake, a ketogenic metabolic state may be achieved [5].

Numerous health benefits are attributed to the CD on social media, including improvements in chronic diseases (e.g., diabetes, dyslipidemia, hypertension, gastrointestinal conditions), weight reduction, and enhanced physical and cognitive performance [2,4,6]. A central argument frequently put forward is the elimination of so-called antinutritional compounds (AN) found in plant-based foods. These include secondary plant metabolites such as enzyme inhibitors, lectins, phytic acid, oxalic acid, tannins, goitrogens, and saponins, which can impair the bioavailability of certain nutrients and may exert potentially toxic effects [7]. Although the content of these compounds can be reduced through various processing techniques [7,8], they are entirely excluded from the CD due to the complete omission of plant foods. While this may prevent possible interference with nutrient absorption, it simultaneously results in a reduced intake of health-promoting phytochemicals, dietary fiber, and essential micronutrients [9]. The current scientific evidence on the CD is limited. Early case studies—such as those by Vilhjalmur Stefansson [10]—as well as initial observational studies suggest potential health benefits but also indicate risks, including elevated low-density-lipoprotein (LDL-) cholesterol and triglyceride (TG) levels [2] or potential calcium deficiency [10]. Overall, a high intake of animal-source foods, particularly red and processed meat, has been associated with an increased risk of cardiovascular diseases, certain cancers, and reduced life expectancy [11,12,13,14,15,16]. To date, no systematic data exists regarding the short- and long-term health effects of the CD.

The advantages promoted by proponents of the CD—including its elimination-based approach, the potential induction of ketosis, and a purported increase in nutrient bioavailability [5]—stand in contrast to established dietary recommendations, which advocate for a balanced, predominantly plant-based mixed diet for healthy adults [17]. The World Health Organization (WHO) recommends a diverse diet rich in legumes, nuts, and whole grains, along with a daily intake of at least 400 g of fruits and vegetables [18]. A comparison of the Dietary Reference Values (DRVs) issued by the European Food Safety Authority (EFSA) with the nutrient composition of animal-source foods [19] highlights several micronutrients that may be insufficient within the CD, particularly vitamin C, vitamin E, folate, calcium, magnesium, manganese, iodine, and fluoride. However, no standardized definition of the CD currently exists, so actual nutrient intake largely depends on individual food selection. The additional, sometimes practiced avoidance of dairy products may increase the risk of inadequate iodine and calcium intake, while excluding seafood can potentially lead to deficiencies in iodine, vitamin E, and magnesium. Shellfish and mollusks are important sources of magnesium and vitamin E; eggs provide vitamin E and folate; and liver contains relevant amounts of folate and vitamin C [19]. However, due to its high retinol content, liver should only be consumed in limited quantities [20]. In general, the more food groups that are excluded from the diet, the greater the risk of nutrient deficiencies [21]—a principle that applies to all restrictive dietary patterns, regardless of whether they are animal- or plant-based [22,23,24,25].

To evaluate the current state of research regarding the potential benefits and risks of the CD, a scoping review was conducted. The aim was to assess the current state of literature on the health effects and nutritional adequacy of CD, with the objective of informing future research and clinical practice in this field.

## 2. Materials and Methods

The systematic literature search was conducted in accordance with the guidelines of the Preferred Reporting Items for Scoping Reviews (PRISMA-ScR) [26] and the methodological recommendations for scoping reviews by Elm et al. (2019) [27]. Searches were performed in the databases PubMed, LIVIVO, Web of Science, and the Cochrane Library registry. For each database, a tailored search string was developed (see Appendix A; Table A1). In addition, a narrative literature search was carried out to identify further relevant sources beyond those retrieved from the primary databases and registries. All identified publications were imported into the reference management software Citavi 6.19, where duplicates were removed. A two-stage screening process was then applied: first, titles and abstracts were screened, and non-relevant records were excluded. The remaining articles were subsequently assessed in full text, analyzed, and the relevant information was extracted and synthesized. Prior to initiating the search, inclusion and exclusion criteria were defined (Table 1). These criteria were intentionally formulated broadly to ensure that all potentially relevant scientific documents concerning the CD could be captured.

## 3. Results

**Figure 1:**
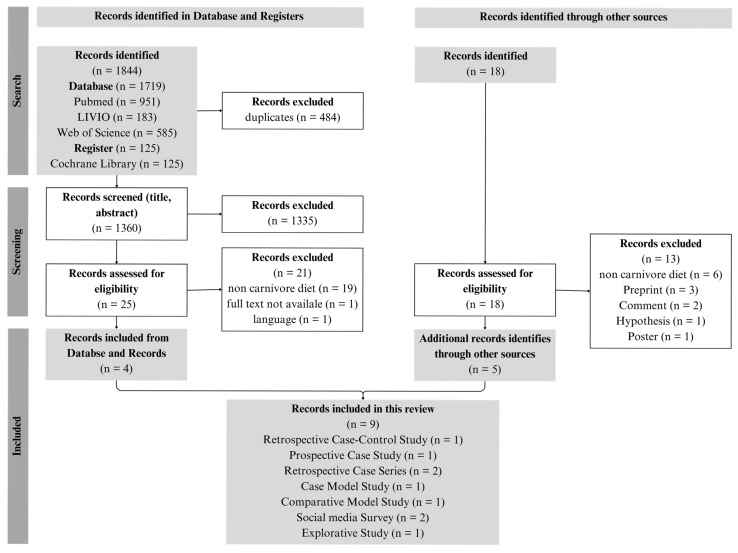
PRISMA Flowchart of the Literature Search.

The literature search was conducted in November 2025. In total, nine English-language studies published between 2021 and 2025 were included in the analysis (Figure 1). Five publications originated from Europe [25,28,29,30,31] and four from the United States [2,32,33,34]. Among the identified publications were five case studies, including one case-model study [25], one prospective case study [32], one retrospective case–control study [28], and two retrospective case series [29,34]. In addition, two social media surveys [2,33], one exploratory study [31], and one comparative modeling study [30] were included.

The CD was defined and implemented differently across the included studies (Table 2). With the exception of three publications [2,33,34], all studies excluded plant-based foods entirely. In four studies, the focus of food selection was explicitly on red meat [2,25,30,32]; in two of these, the consumption of high-fat meat was emphasized [25,30], whereas one study primarily involved lean meat consumption [28]. In the case series by Norwitz and Soto-Mota (2024), the CD was initially used as an elimination diet for two participants; subsequently, they transitioned to a ketogenic, meat-centered dietary pattern, with specific attention to adequate salt intake [29]. Another publication explicitly examined a ketogenic variant of the CD [32]. In the social media survey by Protogerou (2021), participants reported following a zero-carbohydrate diet with predominant consumption of meat and organ meats [33]. In addition to meat as the primary dietary component, fish [25,33], organ meats [2,25,33], seafood [2,33], eggs [2,28,29,31,33], and dairy products [2,25,28,29,31,33] were included or considered part of the CD. In one case study and one case within the case series, the diet was restricted exclusively to beef [29,32].

The aims, research questions, and focal points of the included publications varied considerably (Table 2). Two studies developed theoretical dietary plans and evaluated them based on achieved nutrient intakes in comparison with DRVs or the Healthy Eating Index (HEI) [25,30]. Three publications examined the effects of a CD on gynecological, gastrointestinal, and urological conditions [29,32,34]. Three additional studies assessed dietary behaviors, health status, and anthropometric and sociodemographic characteristics using self-developed questionnaires [2,31,33], with two of these also documenting laboratory parameters and medication use [2,31]. Furthermore, one study compared the effects of a CD with those of an omnivorous diet regarding the diversity and functionality of the intestinal microbiome [28]. Following completion of the screening process, the following thematic focus areas were identified: motivations for adopting a CD [2,31,33]; laboratory analyses (blood and urine markers) [2,28,29,31,34]; nutrient composition analyses [25,30]; microbiome diversity and functionality [28]; changes in health status [2,29,31,32,33]; reduction or discontinuation of medication [2,29]; and weight changes [2,29].

In two of the three studies examining potential micronutrient deficiencies associated with the CD [5,25,30], several nutrients were classified as insufficient compared with the DRVs, with deficiencies varying by sex and food selection. Reported intakes fell below the DRVs for thiamine (male DRV: 1.2 mg, CD: 0.52–0.92 mg; female DRV: 1.1 mg, CD: 0.64–0.77 mg), vitamin C (male DRV: 45 mg, CD: 1.21–33.2 mg; female DRV: 45 mg, CD: 1.22–16.8 mg), vitamin D, magnesium (male DRV: 400–420 mg, CD: 188.3–203.1 mg; female DRV: 310–320 mg, CD: 135.8–201.8 mg), iron (female DRV: 18 mg, CD: 10.93–19.8 mg), iodine (female DRV: 150 μg, CD: 105.3–908.8 μg), potassium, and calcium (male DRV: 1000 mg, CD: 76.2–764.4 mg; female DRV: 1000 mg, CD: 186.8–840.1 mg). Furthermore, dietary fiber intake was found to be far below the recommended Adequate Intake (AI) (AI male/female: 30/25 g; CD: <1 g). Vitamin A, by contrast, substantially exceeded recommended amounts (male DRV: 900 μg, CD: 1323.4–42,997.4 μg; female DRV: 700 μg, CD: 1046.6–26,320.2 μg) [25,30].

Across four studies, positive effects on various disease courses were reported, in some cases enabling a reduction or discontinuation of medication [2,29,31,32]. Lennerz et al. (2021) observed improvements under a CD in several laboratory parameters, including C-reactive protein (CRP) (1.0 mg/dL vs. 0.7 (0.8) mg/dL, *p* < 0.01), gamma-glutamyltransferase (GGT) (18 (19) U/L vs. 15 U/L, *p* < 0.01), and the triglyceride (TG) to high density lipoprotein cholesterol (HDL-C) ratio (TG: 83 mg/dL vs. 68 mg/dL, *p* < 0.01; HDL-C: 58 mg/dL vs. 68 mg/dL, *p* < 0.01) [2]. According to Norwitz and Soto-Mota (2024), individuals with inflammatory bowel disease (IBD) exhibited substantial reductions in fecal calprotectin (e.g., 3300 μg/g vs. 870 μg/g; 4291 μg/g vs. 9 μg/g) and improvements in iron status following anemia [29]. Conversely, increases in platelet counts, LDL-C (126 mg/dL vs. 172 mg/dL, *p* < 0.01), and total cholesterol (TC) (209 mg/dL vs. 256 mg/dL, *p* < 0.01) were reported in the same studies [2,29]. In Klement and Matzat (2025) [31], participants with pre-existing metabolic disorders showed improvements in hemoglobin A1c (HbA1c) and triglycerides, whereas these parameters increased in participants who had been metabolically healthy prior to initiating the CD. Both TC (pre-diet median: 224 mg/dL; on CD: 305 mg/dL; *p* < 0.0001) and LDL-C (pre-diet: 157 mg/dL; on CD: 256 mg/dL; *p* = 0.00024) increased significantly. Overall, many blood values in the cohort remained outside their respective reference ranges, although the proportion decreased slightly compared with pre-diet status (pre-diet: 64/262 [24.4%], on CD: 48/262 [18.3%]; χ^2^ test: *p* = 0.11) [31]. In the case report by Wilson and Moe (2025) [34], the patient’s health deteriorated under the CD. The 24 h urine profile shifted unfavorably (calcium: 129 mg vs. 181 mg; oxalate: 35 mg vs. 47 mg; uric acid supersaturation: 0.54 vs. 1.14; citrate: 342 mg vs. 303 mg; sodium: 65 mmol vs. 235 mmol; potassium: 125 mL vs. 80 mL). The patient required an increased allopurinol dosage of 300 mg/day to manage recurrent gout flares. After discontinuing the CD, the patient remained free of kidney stones one year later [34]. One study found no significant differences in gut microbiome diversity or functionality when comparing a CD with an omnivorous diet [28].

The primary motivation for adopting the CD was reported to be health improvement, followed by perceptions of naturalness and, to a lesser extent, ethical considerations. Many adherents described enhancements in their overall health, as well as in physical and cognitive performance; they also emphasized the diet’s simplicity and the positive sensory experience associated with consuming animal-based foods [2,31,33,34]. Protogerou (2021) further demonstrated that individuals following a Zero-Carb/CD lifestyle frequently encounter social conflict outside the CD community, including tensions within family and friendship networks as well as challenges in interactions with healthcare professionals [33].

## 4. Discussion

Overall, the available evidence presents a heterogeneous picture, characterized by substantial variability in how the CD is implemented across studies and the National Health and Medical Research Council (NHMRC) levels of evidence are predominantly low (Levels III–IV) [35]. A potential advantage of the CD lies in the high nutrient density and biological value of animal-source foods. These provide essential micronutrients and proteins in forms that are generally absorbed and metabolized more efficiently by the human body than their plant-based counterparts, including retinol [36], heme iron [37], and essential amino acids [38]. The complete exclusion of plant foods eliminates AN compounds such as lectins, phytic acid, and tannins, which may increase the bioavailability of several nutrients, including proteins, zinc, iron, calcium, magnesium, phosphorus, manganese, iodine, and vitamins A, E, and D [7,39,40,41,42,43,44]. Moreover, meat contains various bioactive compounds—so-called “carninutrients”—such as creatine, L-carnitine, taurine, and coenzyme Q10, which play important roles in physiological processes and may exert health-promoting effects [45]. Despite this high nutrient density, current evidence suggests that the CD does not meet all recommended DRVs for essential micronutrients. In addition to insufficient intakes of potassium, calcium, vitamin C, vitamin D, thiamin, magnesium, iron, folate, and iodine [25,30], nutrients that occur only in small amounts in animal-source foods—such as vitamin E, manganese, and fluoride—may also be of concern [19]. Depending on how strictly the CD is defined, the exclusion of additional foods such as dairy products or organ meats may further increase the risk of micronutrient deficiencies. Lennerz et al. (2021) reported that 37% of participants did not use vitamin supplements [2]. Similarly, Protogerou et al. (2021) noted that supplement use was generally uncommon (7.56%), although some participants reported taking vitamins/minerals, collagen powder, and encapsulated organ-meat products [33]. Potential nutrient shortfalls (e.g., vitamin C and certain B vitamins) and the absence of readily available carbohydrates as a rapid energy source may contribute to perceived weakness and fatigue [46,47], particularly during the adaptation phase. In addition, inadequate magnesium intake may predispose to muscle tension and cramps, as magnesium helps regulate calcium influx into muscle cells; deficiency can facilitate excessive intracellular calcium and neuromuscular irritability [48]. Many individuals on a CD increase salt intake, because reduced carbohydrate intake lowers insulin and glycogen-associated water retention, promoting natriuresis and fluid loss [49]. If sodium and fluid are not replaced, electrolyte and volume depletion can lead to symptoms commonly described as “keto flu” (e.g., headaches, dizziness, fatigue) [50,51,52] and may increase the risk of orthostatic hypotension, especially early on while cardiovascular regulation adapts to the reduced plasma volume [53,54]. O’Hearn (2020) [5] hypothesized that strict adherence to a CD might alter requirements for certain micronutrients, particularly vitamin C, due to metabolic adaptations. These adaptations could be related to reduced carbohydrate metabolism, shifts in the gut microbiome, and increased intake of animal products. Although meat contains very little vitamin C, no clinical cases of scurvy associated with CD adherence have been reported to date. O’Hearn attributes this to potential antiscorbutic properties of meat and the possible compensatory role of L-carnitine [5]. However, this hypothesis remains speculative and requires empirical validation. It is well established that human metabolism can adapt to different dietary patterns. For example, plant-based diets promote increased absorption of non-heme iron [55] and enhanced expression of carnitine transporters [56], thereby improving nutrient uptake. Carbohydrate restriction, in contrast, can shift the body’s primary energy source from glucose toward ketone bodies [57]. Comparable adaptation mechanisms may also occur under a CD, but they have not yet been systematically investigated.

Another critical aspect of the CD concerns its high proportion of saturated fatty acids. Nutrient analyses by Goedecke et al. (2024) [25] demonstrate that saturated fat intake in CD meal plans substantially exceeds the WHO’s recommended upper limit of <10 E% of total energy intake. Such elevated consumption may increase the long-term risk of coronary heart disease (CHD) [18,25,58]. Several studies further report increases in TG and LDL-C among individuals adhering to a CD [2,29,31]. Current evidence suggests that the ratio of LDL-C to Apolipoprotein B (ApoB) represents an even more informative biomarker for assessing atherosclerotic cardiovascular disease (ASCVD) risk [59,60]. Case-based evidence indicates that physiological lipid profiles may persist despite high fat intake in isolated individuals. For instance, the participant examined by Karačić et al. (2024) exhibited normal cholesterol levels, although creatinine, uric acid, and creatine kinase were mildly elevated—findings consistent with an overall healthy clinical classification [28]. A meta-analysis on red meat consumption found that moderate intake (≈160 g/day) exerts only minor effects on LDL-C and other cardiovascular risk markers [61]. However, it remains unclear whether the very high meat intakes reported in some CD cohorts—up to 1500 g/day in Klement and Matzat (2025) [31]—and the associated lipid changes translate into an increased cardiovascular risk, given the limited quality and quantity of available data. Notably, individuals with pre-existing metabolic conditions showed improved lipid and glucose-related markers in this study, whereas metabolically healthy participants experienced increases in TG, HbA1c, TC, and LDL-C while following the CD [31]. Across the included studies, measured lipid values frequently exceeded common reference ranges [2,29,31,62], with the exception of individuals not exhibiting the so-called lean mass hyper-responder phenotype, in whom elevated LDL-C occurs despite otherwise favorable lipid markers [63]. In this context, the extremely low fiber intake characteristic of the CD is of particular relevance [25]. Dietary fiber is recognized as a key preventive factor for cardiometabolic diseases [64,65]. Adequate fiber intake is associated with reductions in TC and LDL-C, improved postprandial glucose and insulin responses [7], and is therefore recommended in the prevention and management of diabetes [66,67] and CHD [68]. In addition, fiber supports normal gastrointestinal function by increasing stool volume and reducing intestinal transit time [69]. The WHO recommends a daily intake of at least 25 g for adults [64], whereas the CD provides far below this amount, often <1 g/day, as shown in the analysis by Goedecke et al. (2024) [25]. Similarly, high sodium intake—reported in some CD versions [30]—may increase the risk of hypertension and associated cardiovascular and renal diseases [70]. The CD also lacks bioactive phytochemicals with demonstrated antidiabetic [71], antioxidant [72,73], anti-inflammatory [74], and anticancer [75] properties. Leonard (2020) hypothesized that a potentially elevated cancer risk associated with high meat consumption may be offset by metabolic benefits of ketosis and weight loss; however, no empirical evidence currently supports this claim [57]. In contrast, a systematic review by Palmer (2025) [12] concluded that the long-term adverse effects of red and processed meat consumption—such as increased risks of type 2 diabetes, cancer, obesity, hypertension, inflammation, and atherosclerosis—clearly outweigh any potential benefits [12]. Epidemiological data consistently show that low consumption of whole grains, fruits, nuts, and fish combined with high intake of meat products is associated with greater risk of diet-related chronic diseases and increased disability-adjusted life years (DALYs) [13,14,15].

Some variants of the CD emphasize the consumption of unprocessed meat with particular attention to animal quality and production methods, which can substantially influence the nutritional profile of the diet. For example, meat from grass-fed animals generally contains higher concentrations of omega-3 fatty acids and exhibits a more favorable omega-6/omega-3 ratio compared with conventionally raised livestock [76,77]. In this context, the traditional diet of the Inuit is frequently cited in discussions of animal-based dietary patterns. Their diet consisted predominantly of raw animal-source foods—such as meat and blubber from whales, seals, and polar bears—as well as caribou meat [78,79]. Despite its high fat content, this dietary pattern was associated with generally good health outcomes, largely due to its favorable fatty acid composition and substantial intake of essential micronutrients [80,81]. Likewise, the health status of the Maasai—semi-nomadic pastoralists in southern Kenya and northern Tanzania whose subsistence is based on cattle, sheep, and goats [82]—is often referenced in support of meat-based diets [83,84]. However, their low rates of CHD [85,86] cannot be attributed solely to their traditional dietary practices. Instead, they arise from a complex interplay of factors, including genetic characteristics influencing cholesterol metabolism [87,88,89], high levels of physical activity [85,86], chronic caloric restriction, and intermittent fasting [90,91,92]. At the same time, negative aspects of the Maasai dietary pattern have been documented, including micronutrient deficiencies [93,94], a high prevalence of anemia [85,90], and an overall shorter life expectancy [85,95]. These considerations underscore the limited transferability of their health outcomes to modern populations, which are typically far less physically active. Furthermore, the consumption of raw meat and dairy products carries substantial microbiological risks, particularly from pathogens such as *Salmonella* spp. and *Listeria monocytogenes* [31,96]. To date, no scientific evidence supports health benefits of raw animal-product consumption, whereas the risk of infection is well established [96]. Individuals with compromised immune systems and young children are particularly vulnerable, as infections with pathogenic microorganisms can lead to severe clinical outcomes [97].

Given its high proportion of fat and protein alongside very low carbohydrate intake, the CD can be classified as a LCHF dietary pattern that may induce a ketogenic metabolic state. Ketogenic diets have been associated in the literature with various health benefits, including weight reduction and improvements in glycemic control, inflammatory markers, and overall metabolic health [98,99,100,101,102]. The favorable metabolic outcomes reported in previous CD studies may therefore be attributable, at least in part, to its ketogenic characteristics. A notable example is the case series by Norwitz and Soto-Mota (2024), in which seven patients with IBD achieved clinical remission while following a ketogenic CD; in several cases, medication use was reduced or discontinued, and body weight decreased [29]. These effects may be explained by the anti-inflammatory actions of β-hydroxybutyrate (β-HB) on immune cells [103,104]. Similar observations were made in a case study by Yar et al. (2022) [32], in which a ketogenic CD led to remission in a patient with Candida vulvovaginitis and hidradenitis suppurativa. The authors suggested that ketosis may shift energy availability in favor of host cells while exerting anti-inflammatory effects [32]. Potential benefits of ketosis have also been discussed in the context of migraine. Proposed mechanisms include reduced cerebral glucose dependence, improved mitochondrial function, and anti-inflammatory properties of β-HB [99,100,105]. Stanton (2024) further hypothesized that ketosis may correct electrolyte- and pH-related imbalances associated with migraine attacks [106]. In the survey by Lennerz et al. (2021) [2], participants reported improvements in inflammatory and hepatic parameters (CRP, GGT) as well as an improved TG/HDL-C ratio. Reduced insulin resistance and a lower requirement for antidiabetic medications were also noted [2]. These effects are consistent with well-documented metabolic adaptations to classical ketogenic diets [101,102]. Ketone bodies, particularly β-HB, are also known to influence hunger and satiety regulation, potentially reducing energy intake [107]. However, it remains unclear to what extent participants in the included studies actually achieved a sustained ketogenic state, as high protein intake can inhibit ketogenesis through increased gluconeogenesis [108,109]. Consequently, it is difficult to determine whether participants were following a genuinely ketogenic diet or merely a carbohydrate-restricted (“low-carb”) dietary pattern [110,111].

Despite its potential risks and high degree of restrictiveness, the CD is predominantly adopted by its practitioners for perceived health benefits [2,31,33]. In the large social media survey conducted by Lennerz et al. (2021), most participants reported following the CD with the intention of improving their physical and mental well-being [2]. Similar findings were reported by Klement and Matzat (2025) [31]. This indicates that the CD is generally pursued not for ideological or aesthetic reasons, but rather for subjective therapeutic purposes—often with the hope of alleviating symptoms or enhancing overall performance. The health improvements reported in association with the CD [2,29,31,32] may, at least in part, be attributable to placebo effects. Strong beliefs in the diet’s potential benefits can promote behavioral changes that genuinely improve health. For example, individuals who believe that a vegetarian diet facilitates weight loss demonstrate greater motivation to adhere to such a diet, which in turn can lead to actual weight reduction [112]. Moreover, there is evidence that expectations alone can modulate physiological responses. Studies show that merely believing in the efficacy of a supplement can improve cognitive performance or mood, even when the supplement is in fact inert [113,114]. Likewise, the expectation that satiety enhances concentration can itself improve cognitive performance [115]. Food also carries substantial cultural, social, and moral significance [116,117,118]. In this context, the findings of Protogerou (2021) [33] are particularly noteworthy: individuals adhering to a zero-carbohydrate diet frequently reported social conflict—both within personal relationships and in interactions with healthcare professionals outside their community. Social pressure sometimes led participants to consume plant foods despite their dietary intentions. The study further demonstrated that social interactions across various online platforms had a considerable influence on participants’ dietary behaviors [33]. Beyond health-related motivations, the simplicity of meal preparation may provide an additional incentive for adopting the CD. The markedly limited range of permitted foods reduces the need for complex dietary planning, which can be appealing to individuals with low interest in cooking or dietary variety [119]. Taken together, these motivations for adopting the CD illustrate a tension between the subjective perception of benefit and the potential health risks indicated by scientific evidence. It is important to note that many chronic diseases develop over decades. Therefore, current data are insufficient to assess long-term disease outcomes of the CD, despite claims often made on social media. None of the available studies reported clinical endpoints, and there is a lack of prospective longitudinal cohort data on the CD. Given its nutrient profile, potential deficiencies could plausibly increase the risk of long-term conditions such as cardiovascular disease and impaired bone health. Supporting the need for caution, a long-term (≈25-year) follow-up study reported a U-shaped association between carbohydrate intake and mortality, with higher risk at both very low (<40%) and very high carbohydrate intakes [120]. Although long-term data on KD is also limited, it is generally considered a safe therapy for conditions such as epilepsy. However, KD has also been associated with adverse effects such as slowed growth, kidney stones, and fractures [121], and concerns remain regarding liver health and gut microbiome changes [122,123].

In addition to health-related and nutritional considerations, the ecological implications of the CD must also be taken into account [124]. Owing to its nearly exclusive reliance on animal-source foods, the CD stands in clear contradiction to the principles of sustainable and climate-conscious eating patterns [125]. Numerous studies have demonstrated that high consumption of animal products is associated with substantially increased greenhouse gas emissions and other environmental burdens [126,127,128,129,130,131,132,133]. Comparative analyses of popular dietary patterns further show that meat-intensive diets—such as the Paleo diet or certain ketogenic regimens—have a significantly larger environmental footprint than balanced, plant-forward dietary approaches [134]. Given that the CD typically involves even higher quantities of animal products, it is reasonable to assume that its environmental impact is particularly unfavorable relative to other dietary patterns [125]. A noteworthy aspect in this context is that some individuals adhering to the CD previously followed a vegan diet—thus shifting to an almost diametrically opposed eating pattern. It is equally striking that ethical motivations are frequently cited as a reason for adopting the CD [2,31], even though these motivations do not necessarily align with conventional considerations of animal welfare or environmental sustainability [135].

## 5. Conclusions

The findings of this review indicate that the CD may elicit short-term health improvements in certain individuals. However, the overall body of evidence suggests that these effects are likely driven by subjective perceptions, potential placebo responses, and metabolic adaptations associated with ketosis, rather than by robust scientific validation. Due to its extreme exclusion of plant-derived foods, the CD carries an elevated risk of micronutrient deficiencies, adverse alterations in lipid profiles, extremely low fiber intake, and potential renal and cardiovascular health risks. Moreover, social influences and strong online community structures appear to play a substantial role in dietary adherence, while limited social acceptance and skepticism from healthcare professionals may create conflict. From an ecological standpoint as well, the CD stands in clear opposition to principles of sustainable nutrition.

Given the very limited scientific evidence (NHMRC III–IV), small sample sizes, absence of control groups, and short study durations, it is not currently possible to reliably assess the long-term safety of the CD. Based on the available data, long-term adherence to the CD cannot be recommended.

## Figures and Tables

**Table 1:** Inclusion and Exclusion Criteria of the Literature Search.

Inclusion Criteria	Exclusion Criteria
Studies on the carnivore diet (defined as the predominant consumption of animal-based foods; exceptions: herbs, coffee, alcohol)	Studies including ≥10% of energy intake from plant-based foods
Human studies	Animal and in vitro studies
Publication period: 1970–2025	Unpublished studies, preprints, conference abstracts, book chapters, reviews
Language: English or German	

**Table 2:** Objectives, Methods, Definitions of the Carnivore Diet, Study Samples, and Main Findings of Included Studies.

Study	Objective	Methods	Definition of CD	Sample	Main Findings
Goedecke et al. (2024) [25]	Micronutrient intake across different variants of the CD	Development of four CD meal plans (2 × F, 2 × M), with (1F + 3M) and without (2F + 4M) dairy; micronutrient comparison against NRVs (NHMRC)	Predominantly fatty, unprocessed ruminant meat; smaller amounts of pork, poultry, fish; occasional dairy and liver; macronutrient distribution: fat 70–75 E%, protein 25–30 E%, carbohydrates < 5 E%	No human participants; comparison with NRVs (NHMRC) for healthy adults (19–51 years; BMI 22.5 kg/m^2^)	Compared to NRVs: ↑ intake of A, B_2_, B_6_, B_12_, Na, P, Se, Zn; ↓ intake of B_1_, C, Ca, Mg; depending on plan (1F: ↓ Fe, Fol, I; 1F + 2F: DF (<AI), K (98% < AI); 3M: ↓ I; 4M: ↓ Fol)
Karačić et al. (2024) [28]	Microbiome diversity and functionality in CD vs. OV	CD: Dietary assessments (AD + MeD), laboratory analyses (blood and urine), glucose metabolism monitoring (10 d); CD + OV: 16S rDNA sequencing of stool samples	≥4 years CD; predominantly lean meat, butter, hard cheese, eggs, dairy (cheese and whipped cream); complete exclusion of plant foods	CD: 32-year-old M, former bodybuilder; control cohorts (n = 151 M, 27–37 years) stratified by meat consumption: daily (n = 7), ≥3×/week (n = 68), <3×/week (n = 76)	Laboratory analyses CD: ↑ B12, CK, CORT, Cr, D, Ery, Hb, UA; CD vs. OV: microbiome α- and β-diversity similar; ↑ abundance of Escherichia and Salmonella; minor functional differences in carbohydrate degradation, energy metabolism, K production, inflammatory markers, Hyperacidity, fructose intolerance, intestinal barrier function
Klement & Matzat (2025) [31]	Motivation, dietary behaviors, and health status among CD adherents	Qualitative interviews (30 Min, n = 4), quantitative questionnaires, fasting blood samples before and during CD	≥1 month; mainly meat, eggs, dairy	n = 24 (62.5% M), median age 46 (26–62) years, 67% with chronic diseases; mean CD duration 17 months (median 12 (1–56) months), 62.5% KCD, 17% raw CD; before CD: 33.4% KD, OV 29.2%	Very high meat intake (mean 626 g/day, median 515 g/day, 250–1500 g/day), consumption of organ meats (92%), eggs (75%), dairy (58%), fish (54%), honey (37.5%; 27% KCD); Motivations: health (75%), Curiosity (17%), ethics (4%); subjective improvements in general health, satiety, sleep, mental and physical health; blood samples: ↑ HbA1c, LDL-C, TC, TG, outside of range: 24.4% vs. 18.3%
Lennerz et al. (2021) [2]	Motivation, dietary patterns, health status, and satiety among CD adherents	Social media survey; self-reported health, medication, symptoms, social support, lab values before and during CD	Predominantly red meat (excluding pork), eggs, dairy (excluding milk), organ meat (≥42%/week); smaller amounts of pork, poultry, seafood; minimal plant foods (≤10% consumed more than once/month)	n = 2029 (67% M, 33% F, 0.2% Di), median age 44 (34–54) years, BMI 24.3 (22.1–27.0) kg/m^2^, diverse chronic diseases; CD duration median 14 (9–20) months	Self-reported improvements in general health, satiety, sleep, mental and physical health, chronic diseases; worsening lipid profiles (27%); Lab values: ↑ LDL-C, TC, ↓ CRP, GGT, TG/HDL; reductions in diabetes medication, bodyweight
Norwitz & Soto-Mota (2024) [29]	Treatment outcomes for IBD using KD or CD	Lab analyses, 48 h dietary records, Inflammatory bowel disease quality of life (IBDQ) before/after dietary intervention	Exclusion of plant foods (n = 7); partial restriction of dairy/eggs; intentional high salt intake; CD used as elimination diet (n = 2) CD: median 1 year (4 months–6 years)	CD n = 7 (85.7% M), mean age 34 (30–62) years; Crohn’s disease (n = 4), ulcerative colitis (n = 3)	Clinical remission (n = 7); improved CRP, calprotectin, Fe; ↑ LDL-C and TC; reduction in medication (n = 5), bodyweight (n = 2); IBDQ 95 vs. 216
Phelan et al. (2023) [30]	Nutritional quality of popular fad diets	One-week meal plans; nutrient analyses using HEI	Predominantly fatty animal products; exclusion of plant foods; no calorie restriction	CD, FD, FODMAP, KD, LD, PB/VD, PD; comparison with RDAs for healthy adults (19–50 years)	CD scored lowest on HEI (30/100); Compared to RDA: ↓ intake of Ca, D, DF, K; deficiencies more pronounced in F
Protogerou et al. (2021) [33]	Motivation, dietary behavior, health status, and social acceptance of CD	Social media survey	Predominantly beef (98%), fatty fish (72%), pork (64%), eggs (62%), dairy (56%), poultry (51%), organ meats (40%), seafood (17%); occasional fruit (11%), chocolate (8%), vegetables (5%) in social situations	n = 170 (65.29% M, 34.12% F, 0.59% Di) from 25 countries; mean age 42.8 ± 12.06 years; 9% with mental health disorders	Self-reported improvements in general health, satiety, sleep, mental and physical health; advantage of CD: Simplicity, freedom, taste; drawbacks: low social acceptance
Wilson & Moe (2025) [34]	Case report on nephrolithiasis	24 h urine analyses pre- and post-CD	90% meat/meat products; <1 serving/week raw fruit/vegetables; no dairy; added salt	M, 67 years, nephrolithiasis	↑ SSCaOx, Ca24, Ox24, SSCaP, pH, SSUA, UA24, Na24, Mg24, P24, NH_4_24, Cl24, Sul24, UUN24, PCR; ↓ Cit24, K24; symptoms resolved after discontinuing CD
Yar et al. (2022) [32]	Long-term management of recurrent Candida vulvovaginitis and hidradenitis via KCD	Baseline examination, lab tests, 43-day strict meat-based KD	Zero-carb all-meat KD (70 E% fat, 30 E% protein), mainly beef	F, 29 years, BMI 34.6 kg/m^2^; diagnosed with Candida vulvovaginitis and hidradenitis	Complete symptom remission within 43 days; sustained improvement at follow-up

A = Vitamin A, AD = athlete diet, AI = Adequate Intake, B_1_ = thiamin, B_2_ = riboflavin, B_6_ = Vitamin B_6_, B_12_ = Vitamin B_12_, C = Vitamin C, Ca = Calcium, CD = carnivore diet, CK = creatine kinase, CORT = cortisol, Cr = creatinine, CRP = C-reactive protein, Ca24 = 24 h-Calcium, Cit24 = 24 h-Citrate, Cl24 = 24 h-Chloride, d = day(s), D = Vitamin D, Di = diverse, DF = dietary fiber, Ery = erythrocytes, E% = % energy, F = female, FD = fasting diet, Fe = iron, FODMAP = Fermentable Oligo-, Di-, Mono-saccharides And Polyols, Fol = Folate, GGT = Gamma-Glutamyl Transferase, Hb = hemoglobin, HbA1c = Hemoglobin A1c, HEI = Healthy Eating Index, I = iodine, IBD = inflammatory bowel disease, K = vitamin K, KCD = ketogenic carnivore diet, KD = ketogenic diet, K24 = 24 h-Potassium, LD = liquid diet, LDL-C = low density lipoprotein cholesterol, M = Male, MeD = Mediterranean diet, Mg = Magnesium, Min = Minute(s), MP = meal plan, Mg24 = 24 h-Magnesium, NA = Niacin, NNHMRC = Australian National Health and Medical Research Council and New Zealand Ministry of Health, NRVs = Nutrient Reference Values, Na24 = 24 h-Sodium, NH_4_24 = 24 h-Ammonium, OV = omnivore diet, Ox24 = 24 h-Oxalate, P = phosphorus, PB = plant based diet, PCR = Proteincatabolicrate, PD = paleo diet, P24 = 24 h-Phosphorus, RDA = Recommended Dietary Allowance, rDNA = Recombinant DNA, Se = Selenium, SSCaOx = Calciumoxalate supersaturation, SSCaP = Calciumphosphate supersaturation, SSUA = Uricacid supersaturation, Sul24 = 24 h-Sulfate, TC = total cholesterol, TG = triglyceride, TG/HDL = triglyceride/high density lipoprotein-ratio, UA = uric acid, UA24 = 24 h-Uricacid, UUN24 = 24 h-Urea-nitrogen, VD = vegan diet, Zn = zinc, ↑ = increased, ↓ = decreased.

## Data Availability

No new data were created or analyzed in this study. Data sharing is not applicable to this article.

## Appendix A

**Table A1:** Search strategy, including database, search term, and filter.

Database	Search Term	Filter
PubMed	“meat based” OR “meat diet” OR “carnivore diet” OR carnivor*[MeSH Terms] OR carnivor* [Other Terms] AND human*[MeSH Terms] OR adult [MeSH Terms] OR patients [MeSH Terms] OR persons [MeSH Terms] OR Research Subject [MeSH Terms] OR human (Other Terms) NOT bear [MeSH Terms] OR Caniformia [MeSH Terms] OR Feliformia [MeSH Terms] OR Carnivorous Plants [MeSH Terms] OR bear [Other Terms]	1970–2025, German, English, Humans
LIVIVO	TI = (carnivor* OR “meat based” OR “meat diet” OR “carnivore diet”) AND TI = (human OR adult OR patient OR person OR “research subject”) AND PY = 1970:2025 NOT TI = (predator OR Rodent*)	1970:2025
Web of Science	#1 TI = (“meat based”) or TS = (“meat diet”) or ALL = (“carnivor* diet”) or ALL = (“all meat diet”) or ALL = (“meat only”) or TS = (carnivor*) #2 ((((TS = (human*)) OR TS = (adult*)) OR TS = (patient*)) OR TS = (person*)) OR TS = (“research subject*”) #3 #1 and #2 #4 ((((((((((ALL = (“carnivorous plant*”)) OR ALL = (predator*)) OR ALL = (rodent*)) OR ALL = (bear*)) OR ALL = (wolve*)) OR ALL = (pet*))) OR ALL = (“human-wildlife”)) OR ALL = (canid*)) OR ALL = (felid*)) #5 (#3) NOT #4 and English or German (Languages) and Proceeding Paper or Book Chapters (Exclude – Document Types) and Environmental Sciences or Food Science Technology or Nutrition Dietetics or Agriculture Dairy Animal Science or Behavioral Sciences or Physiology (Web of Science Categories)	English, German, Environmental Sciences, Food Science Technology, Nutrition Dietetics, Agriculture Dairy Animal Science, Behavioral Sciences, Physiology (included) Proceeding Paper, Book Chapters (excluded)
Cochrane library	#1 (“carnivore diet”) or (“meat diet”) or (“meat based”) or (carnivor*):ti,ab,kw #2 Carnivora [MeSH] #3 Carnivory [MeSH] #4 #1 or #2 or #3 #5 (human*) #6 Humans [MeSH] #7 Adult [MeSH] #8 Patients [MeSH] #9 Persons [MeSH] #10 Research Subjects [MeSH] #11 #5 or #6 or #7 or #8 or #9 or #10 #12 Caniformia [MeSH] #13 Feliformia [MeSH] #14 #12 or #13 #15 #4 and #11 not #14	1970–2025

* The asterisk (*) indicates truncation and was used as a wildcard to capture different word endings (e.g., carnivore, carnivorous, carnivory).
